# Strategies to disrupt NKG2A:HLA-E interactions for improved anti-cancer immunity

**DOI:** 10.18632/oncotarget.28610

**Published:** 2024-07-17

**Authors:** Jack G. Fisher, Lara V. Graham, Matthew D. Blunt

**Keywords:** Natural killer (NK) cells, immunotherapy, NKG2A, immune checkpoint blockade, HLA-E

The inhibitory receptor NKG2A, which forms a heterodimer with CD94, is expressed by ~50% of peripheral blood NK cells and is further upregulated following NK cell expansion *ex vivo* [1]. In addition, NKG2A is a late immune checkpoint on CD8+ T cells that is upregulated following repeated antigen stimulation and division [2]. NKG2A has also been detected on exhausted CAR T cells 27 days post-CAR T cell infusion [3] and on CD8+ tumour infiltrating T cells [4–6]. Interestingly, NKG2A marks a subset of innate-like γδ T cells with potent anti-tumour activity [7]. Engagement of NKG2A by its ligand HLA-E leads to the recruitment and activation of phosphatases which inhibit NK cell and T cell activation [8]. HLA-E is highly expressed in multiple cancers compared to healthy tissues [4, 9] and senescent cells have been shown to evade NK and CD8+ T cell immunity via HLA-E [10]. Two studies which employed CRISPR screens in cancer cells identified HLA-E as a critical negative regulator of NK cell: cancer cell interactions [11, 12]. In accordance with this, IFNγ signalling was associated with NK cell resistance due to increased STAT1 activation and enhanced HLA-E expression [11]. This is also evident with the murine homolog of HLA-E, Qa-1b, which was upregulated by inflammatory signals on all cell types tested [13].

In addition to inflammatory signals, we have recently demonstrated that surface expression of HLA-E is increased by lymph node-associated signals IL-4 and CD40L on primary chronic lymphocytic leukaemia (CLL) cells [14]. In accordance with this, HLA-E surface expression was higher on tumour cells that had recently egressed from the lymph nodes of patients with CLL compared to those that had resided in the peripheral blood for longer [14]. High HLA-E expression induced following IL-4 and CD40L stimulation significantly inhibited NKG2A+ NK cell activation against primary CLL cells, indicating that HLA-E expression on tumour cells can differ depending on the location of the tumour cells within patients. Indeed, HLA-E is induced by IFNγ in the tumour microenvironment in multiple myeloma [15]. Two recent studies have also demonstrated that HLA-E can protect circulating tumour cells from NK cell lysis via NKG2A, indicating that targeting the NKG2A:HLA-E axis may be useful for preventing metastasises of solid tumours [16, 17]. Together, these studies indicate that the disruption of the NKG2A:HLA-E axis has strong potential to improve immunity against haematological malignancies and solid tumours.

Unlike classical HLA class I proteins, which rapidly exit the endoplasmic reticulum (ER) after synthesis, the non-classical HLA class I protein HLA-E is mostly retained intracellularly due to a limited supply of high-affinity HLA-E-binding peptides [18]. These HLA-E-binding peptides are primarily derived from the leader sequences of classical HLA class I proteins (HLA-A/B/C) with sequence VMAPRTL/VV/L/FL which are collectively named VL9 peptides [19, 20]. Alongside regulating exit of HLA-E from the ER, VL9 peptides are required for plasma membrane stabilisation of HLA-E, as too are Qa-1b-binding peptides (Qdm) in mice [18, 21]. Once at the cell surface, HLA-E can be rapidly internalized and is thought to act as a real-time sensor for optimal peptide loading complex-mediated antigen processing and presentation, as well as HLA class I expression [13, 22]. Disruption of peptide availability, for example by the protein transport inhibitor brefeldin A, rapidly reduces surface HLA-E expression [14, 18, 23]. Importantly for the clinical utility of this effect, approved cancer therapies which disrupt protein synthesis and protein transport such as the exportin-1 inhibitor selinexor and the proteosome inhibitor bortezomib can induce the downregulation of surface HLA-E on tumour cells (Figure 1) and enhance NKG2A+ NK cell activation [14, 23]. On the reverse, the clinically approved tyrosine kinase inhibitor dasatinib, used for the treatment of chronic myeloid leukaemia (CML), has been shown to downregulate NKG2A expression on patient NK cells *in vivo*, resulting in enhanced NK cytotoxicity against CML cell lines *ex vivo* [24]. This highlights a novel mechanism of action for these drug classes which could potentially be utilised to optimise their combination with immunotherapies. Furthermore, preclinical compounds are also showing evidence of NKG2A:HLA-E disruption, with inhibitors of CREB1 recently shown to sensitise multiple myeloma cells to NK cytotoxicity via HLA-E downregulation [15].

**Figure 1:**
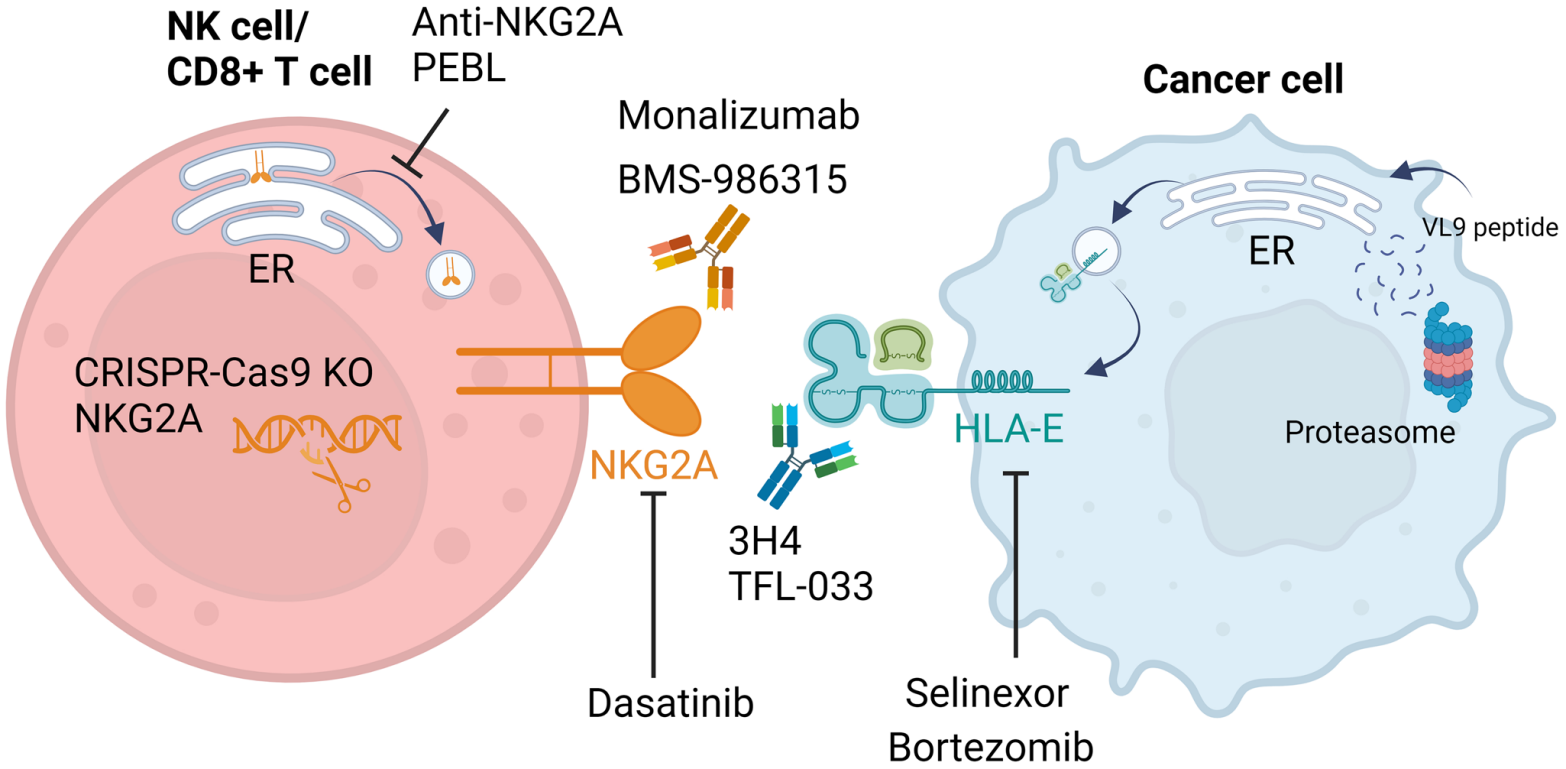
Cancer therapeutics targeting the NKG2A:HLA-E axis. The NKG2A:HLA-E interaction can be inhibited by antibodies targeting either NKG2A (monalizumab, BMS-986315) or HLA-E (3H4, TFL-033). Small molecule inhibitors bortezomib and selinexor have been shown to reduce surface expression of HLA-E, sensitising tumour cells to NK cell cytotoxicity. The proteasome inhibitor bortezomib induces endoplasmic reticulum (ER) stress, potentially leading to impaired peptide loading which is crucial for expression of HLA-E at the cell surface. The precise mechanism behind the downregulation of surface HLA-E by selinexor has not yet been described. Expression of NKG2A on the surface of NK cells can also be manipulated by CRISPR-Cas9 knock-out (KO), anti-NKG2A protein expression blocker (PEBL) that can retain NKG2A in the ER, and the tyrosine kinase inhibitor dasatinib that can induce NKG2A downregulation. Overall, either blockade or downregulation of NKG2A and HLA-E relieves NK cell inhibition and improves lysis of tumour cell targets. Created with https://www.biorender.com/.

Disruption of NKG2A:HLA-E interactions can also be achieved by blocking antibodies targeting either NKG2A or HLA-E. NKG2A blocking antibodies promote NK cell and CD8+ T cell effector functions in preclinical tumour models [4]. Furthermore, intratumoural injection of NK cells and the NKG2A blocking antibody monalizumab improved tumour regression in solid tumour mouse models [25]. The most clinically advanced NKG2A blocking antibody monalizumab is currently being assessed in a phase 3 clinical trial in combination with durvalumab in patients with locally advanced (Stage III), unresectable Non-small Cell Lung Cancer (NSCLC) (NCT05221840). During the phase 2 COAST trail of 186 patients with NSCLC, durvalumab in combination with monalizumab obtained an objective response rate of 40.3% compared to durvalumab single agent treatment (23.9%), with increased median duration of response (difference of 9 months) and increased 2-year overall survival (72.1% combination vs 61.5% durvalumab) [26]. In terms of adverse events, the frequency and severity were comparable between treatment groups [26]. In addition, the NKG2A blocking antibody BMS-986315 plus nivolumab in combination with platinum-based doublet chemotherapy is currently in a phase 2 clinical trial for Stage IV or recurrent NSCLC (NCT06094296). Furthermore, HLA-E blocking antibodies have been shown to potentiate NK cell activation [27, 28], indicating that, at least in preclinical models, this checkpoint can be targeted at both the ligand or receptor level, similar to the PDL-1/PD-1 checkpoint. Interestingly, VL9 peptides derived from different HLA class I molecules influenced the ability of NKG2A blocking antibodies to mediate NK specific lysis of tumour cells [29]. In addition to antibody blockade, silencing of NKG2A expression in NK cells using CRISPR-Cas9 technology [30] and protein expression blockers [9] have improved NK lysis of tumour cells in preclinical models, demonstrating the potential of these methods to improve anti-tumour immunity. Although, caution should be applied with knock-out approaches given the recent evidence that NKG2A is required for maintenance of NK expansion capabilities [31] and for NK cell education [32].

In conclusion, there is strong preclinical evidence that disruption of NKG2A interactions with HLA-E can stimulate both NK cell and cytotoxic T cell effector functions against cancer. The results from the phase 3 clinical trial of the NKG2A blocking antibody monalizumab are eagerly awaited. In addition, the downregulation of HLA-E by the approved anti-cancer drugs selinexor and bortezomib may allow for their optimal combination with immunotherapies. Given that HLA-E is overexpressed in multiple cancer types, disruption of NKG2A:HLA-E interactions has the potential to enhance anti-cancer immunity and improve patient outcomes in multiple clinical settings.

## References

[1] Fisher JG , et al. Vaccines (Basel). 2022; 10:1993. 10.3390/vaccines10121993. 36560403 PMC9783329

[2] Borst L , et al. Int J Cancer. 2022; 150:688–704. 10.1002/ijc.33859. 34716584 PMC9299709

[3] Good CR , et al. Cell. 2021; 184:6081–100.e26. 10.1016/j.cell.2021.11.016. 34861191 PMC8827167

[4] André P , et al. Cell. 2018; 175:1731–43.e13. 10.1016/j.cell.2018.10.014. 30503213 PMC6292840

[5] van Montfoort N , et al. Cell. 2018; 175:1744–55.e15. 10.1016/j.cell.2018.10.028. 30503208 PMC6354585

[6] Qiu Y , et al. J Immunother Cancer. 2024; 12:e008368. 10.1136/jitc-2023-008368. 38262706 PMC10824007

[7] Cazzetta V , et al. Cell Rep. 2021; 37:109871. 10.1016/j.celrep.2021.109871. 34686325

[8] Le Dréan E , et al. Eur J Immunol. 1998; 28:264–76. 10.1002/(SICI)1521-4141(199801)28:01<264::AID-IMMU264>3.0.CO;2-O. 9485206

[9] Kamiya T , et al. J Clin Invest. 2019; 129:2094–106. 10.1172/JCI123955. 30860984 PMC6486333

[10] Pereira BI , et al. Nat Commun. 2019; 10:2387. 10.1038/s41467-019-10335-5. 31160572 PMC6547655

[11] Sheffer M , et al. Nat Genet. 2021; 53:1196–206. 10.1038/s41588-021-00889-w. 34253920

[12] Dufva O , et al. Immunity. 2023; 56:2816–35.e13. 10.1016/j.immuni.2023.11.008. 38091953

[13] Middelburg J , et al. Cell Rep. 2023; 42:113516. 10.1016/j.celrep.2023.113516. 38048225

[14] Fisher JG , et al. Leukemia. 2023; 37:2036–49. 10.1038/s41375-023-01984-z. 37528310 PMC10539165

[15] Ismael A , et al. Leukemia. 2024. [Epub ahead of print]. 10.1038/s41375-024-02303-w. 38902472 PMC11286514

[16] Liu X , et al. Cancer Cell. 2023; 41:272–87.e9. 10.1016/j.ccell.2023.01.001. 36706761

[17] Liu X , et al. Cell Discov. 2024; 10:16. 10.1038/s41421-024-00646-3. 38336855 PMC10858264

[18] He W , et al. J Exp Med. 2023; 220:e20221941. 10.1084/jem.20221941. 37140910 PMC10165540

[19] Borrego F , et al. J Exp Med. 1998; 187:813–18. 10.1084/jem.187.5.813. 9480992 PMC2212178

[20] Braud VM , et al. Nature. 1998; 391:795–99. 10.1038/35869. 9486650

[21] Kambayashi T , et al. J Immunol. 2004; 172:1661–69. 10.4049/jimmunol.172.3.1661. 14734748

[22] Cassidy SA , et al. Front Immunol. 2014; 5:133. 10.3389/fimmu.2014.00133. 24744756 PMC3978238

[23] Carlsten M , et al. Oncoimmunology. 2019; 8:e1534664. 10.1080/2162402X.2018.1534664. 30713790 PMC6343814

[24] Chang MC , et al. Front Immunol. 2018; 9:3152. 10.3389/fimmu.2018.03152. 30705677 PMC6344416

[25] Melero I , et al. EMBO Mol Med. 2023; 15:e17804. 10.15252/emmm.202317804. 37782273 PMC10630884

[26] Aggarwal C , et al. Am Soc Clin Oncol. 2024; 42:8046. 10.1200/JCO.2024.42.16_SUPPL.8046.

[27] Li D , et al. Commun Biol. 2022; 5:271. 10.1038/s42003-022-03183-5. 35347236 PMC8960791

[28] Ravindranath MH , et al. Monoclon Antib Immunodiagn Immunother. 2019; 38:38–59. 10.1089/mab.2018.0043. 31009335 PMC6634170

[29] Battin C , et al. Immunology. 2022; 166:507–21. 10.1111/imm.13515. 35596615 PMC9426624

[30] Bexte T , et al. Oncoimmunology. 2022; 11:2081415. 10.1080/2162402X.2022.2081415. 35694192 PMC9176243

[31] Kaulfuss M , et al. Sci Rep. 2023; 13:10555. 10.1038/s41598-023-37779-6. 37386090 PMC10310841

[32] Zhang X , et al. Nat Commun. 2019; 10:5010. 10.1038/s41467-019-13032-5. 31676749 PMC6825122

